# Local administration of a novel Toll-like receptor 7 agonist in combination with doxorubicin induces durable tumouricidal effects in a murine model of T cell lymphoma

**DOI:** 10.1186/s13045-015-0121-9

**Published:** 2015-03-04

**Authors:** Jiang Zhu, Shiping He, Jie Du, Zhulin Wang, Wang Li, Xianxiong Chen, Wenqi Jiang, Duo Zheng, Guangyi Jin

**Affiliations:** Shenzhen Key Laboratory of Translational Medicine of Tumor, School of Medicine, Shenzhen University, 3688 Nanhai Avenue, Shenzhen, 518060 People’s Republic of China; Department of Medical Oncology, Sun Yat-Sen University Cancer Center, 651 Dong Feng RD East, Guangzhou, 510060 People’s Republic of China; Shenzhen Engineering Lab of Synthetic Biology, School of Medicine, Shenzhen University, 3688 Nanhai Avenue, Shenzhen, 518060 People’s Republic of China

**Keywords:** Toll-like receptor 7, Doxorubicin, Immunotherapy, Lymphoma

## Abstract

**Background:**

Conventional chemotherapy and radiotherapy for the treatment of lymphoma have notable drawbacks, and passive immunotherapy using a monoclonal antibody is restricted to CD20-positive B cell lymphoma. Therefore, new treatment types are urgently required, especially for T cell lymphoma. One type of new antitumour therapy is the use of active immunotherapeutic agents, such as agonists of the Toll-like receptors (TLRs), which facilitate the induction of prolonged antitumour immune responses.

**Methods:**

We have synthesised a novel TLR7 agonist called SZU-101 and investigated the systemic antitumour effect on a murine model of T cell lymphoma *in vivo*.

**Results:**

Here, we report that the intratumoural administration of SZU-101 enhanced the effectiveness of a conventionally used chemotherapeutic agent, doxorubicin (DOX). SZU-101 administration improved tumour clearance in a murine model of T cell lymphoma. The novel combination of intratumourally administered SZU-101 and DOX generated strong cytokine production and enhanced the cytotoxic T lymphocyte response, leading to the eradication of both local and distant tumours in tumour-bearing mice.

**Conclusions:**

These findings suggested that combined active immunotherapy can be developed as a promising treatment for T cell lymphoma, which may further improve the effectiveness of the current standard cyclophosphamide, DOX, vincristine and prednisone (CHOP) therapy.

**Electronic supplementary material:**

The online version of this article (doi:10.1186/s13045-015-0121-9) contains supplementary material, which is available to authorized users.

## Background

Traditional treatments for lymphoma, such as chemotherapy and radiotherapy, have notable drawbacks, and passive immunotherapy using a monoclonal antibody is restricted to CD20-positive B cell lymphoma. In addition, resistance to the treatment is also observed [1]. Therefore, new treatment types are urgently required. One such novel therapy is treatment with active immunotherapeutic agents, such as agonists of the TOLL-like receptors (TLRs), which facilitate the induction of prolonged antitumour immune responses.

TLRs are expressed on a series of immune cells, such as dendritic cells, macrophages, B cells, T cells and natural killer cells, and they recognise specific pathogen-associated molecular patterns (PAMPs). With the exception of TLR3, most TLR signalling depends on myeloid differentiation primary-response protein 88 (MYD88). The activation of MYD88-dependent signalling results in the activation of NF-κB, IFN regulatory factors (IRFs) and activator protein 1 (AP-1), leading to the release of proinflammatory cytokines and stimulatory molecules for the activation of immune cells [2]. As the bridge between innate immunity and adaptive immunity, TLR signalling regulates cytokine production, T helper and effector cells as well as suppressor cells, such as regulatory T cells (Tregs) and myeloid-derived suppressor cells (MDSCs) [3-6].

Recent studies have focused on TLR7, 8 and 9, which are localised intracellularly to the endosomal membranes. TLR7 activation by ligands, such as viral RNA or synthetic agonists, induces strong T_H_-1-biassed immune responses and thus leads to a durable tumouricidal effect by supporting the activation of CD8+ T cells [7]. In addition to the eradication of large primary tumours, the combined application targeting TLR7, 8 and 9 also established long-term antitumour immunity [6]. For the treatment of lymphoma, Jonathan and colleagues established a phase II clinical study in which a TLR9 agonist (1018 ISS) was used with rituximab for follicular lymphoma [8]. Enhanced antitumour efficacy was also found when radiotherapy was combined with the TLR9 agonist CpG DNA 1826 or the TLR7 agonist R848 [9,10].

In this study, we introduced a novel TLR7 agonist called SZU-101 and sought to investigate its immunogenicity and antitumour effects. We hypothesised that locally or systemically administered SZU-101 in combination with conventional chemotherapeutic agents would induce systemic antitumour immune responses. In addition, as one of the conventionally used chemotherapeutic agents for lymphoma, doxorubicin was selected for the combined treatment. Here, we demonstrated that intratumourally administered SZU-101 enhanced the therapeutic effectiveness of doxorubicin (DOX) through the generation of a systemic immune response. The combined application induced the release of proinflammatory cytokines, facilitated the maturation of dendritic cells and activated B cells and T cells, leading to the eradication of both primary and distant tumours in a murine lymphoma model. This study provides evidence for the translation of the combined active immunotherapy to early phase clinical trials for the treatment of lymphoma.

## Results

### SZU-101 activated dendritic cells, B cells and T cells and induced cytokine production but did not directly affect tumour cells

**Figure 1:**
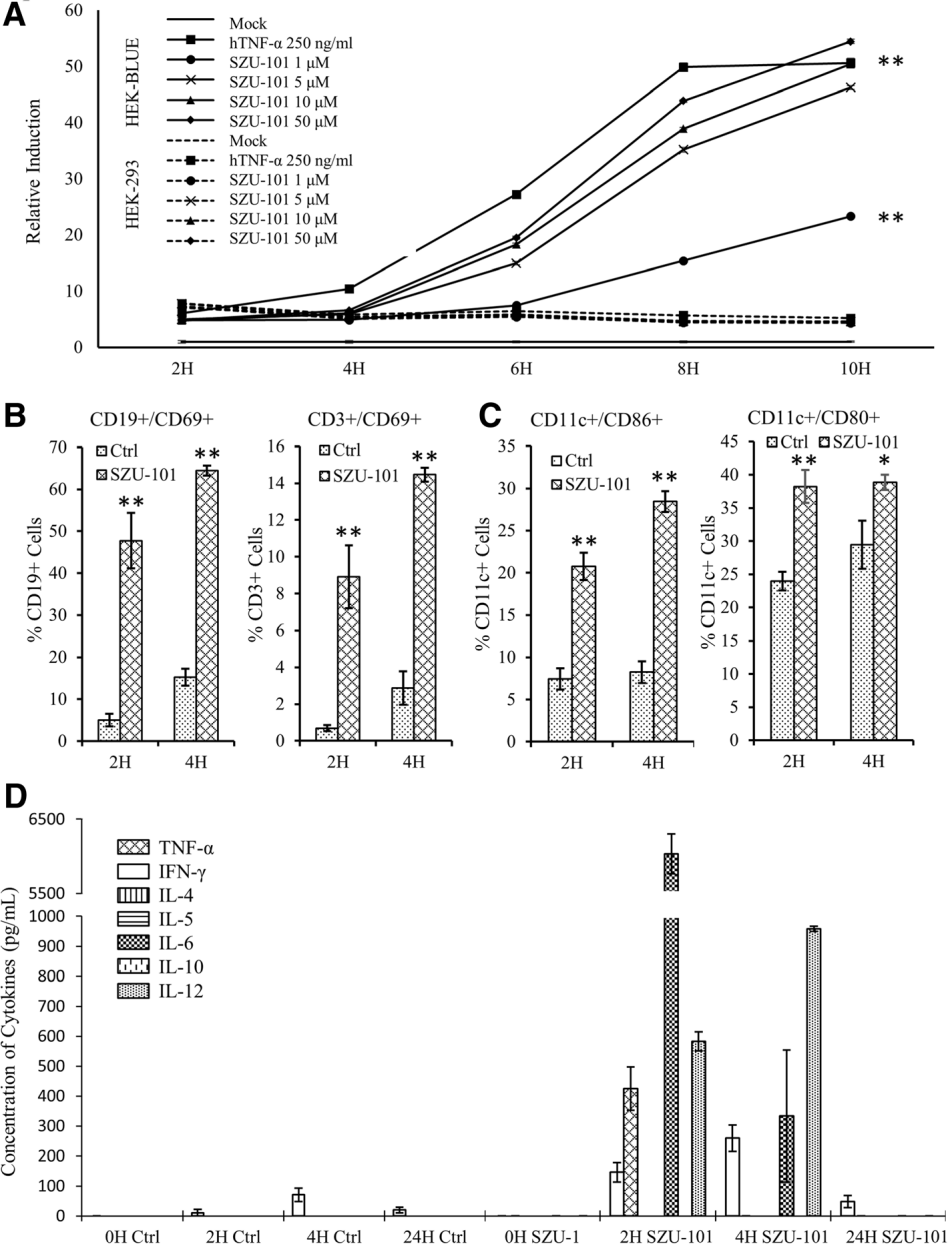
**SZU-101-activated immune response through TLR7 signalling. (A)** SZU-101 induced the activation of TLR7-NF-κB signalling. The solid line indicates the relative inductions by SZU-101 or TNF-α (as positive control) in a HEK-BLUE TLR7-specific reporter system. The dotted line indicates the inductions of SZU-101 or TNF-α in the non-reporter HEK-293 controls (**p < 0.01, one-way ANOVA). **(B)**, **(C)**. The expression of CD69, CD86 and CD80 after SZU-101 stimulation. Splenocytes were collected after 2 or 4 h post SZU-101 treatment and analysed by flow cytometry (**p < 0.01, one-way ANOVA). **(D)** The induction of systemic cytokines was analysed by ELISA assay. The serums were collected after 2, 4 or 24 h post treatment. Each group in the experiment contained five mice.

In this study, we first investigated whether the synthesised SZU-101 compound activated TLR7 signalling and primed the immune response. We introduced a TLR7-NF-kB reporter system expressed in HEK-293 cells and treated the cells with different doses of SZU-101. We found that the reporter was activated after a 4-h treatment and consistently induced reporter activity by 50-fold with higher doses of SZU-101 or up to 23-fold with a low dose of 1 μM SZU-101. The inductions with treatments of more than 5 μM SZU-101 were comparable to those of 250 ng/ml of TNF-α, which directly activates the NF-kB reporter system (Figure 1A). As TLR7 was localised intracellularly to the endosomal membranes, we also treated HEK-293 cells with FITC-labelled SZU-101 and confirmed that SZU-101 could be transported into the cytoplasm (Additional file 1: Figure S1). The result indicated that SZU-101 was a TLR7 agonist that directly activated TLR7 signalling.

To investigate the immune activation effect of SZU-101, splenocytes from SZU-101-treated (3 mg/kg) mice were analysed by flow cytometry. The results revealed that SZU-101 significantly induced the expression of the early activation marker CD69 in both CD3^+^ T cells and CD19^+^ B cells (Figure 1B). We also found that SZU-101 stimulated CD11c + dendritic cells (DCs) to express the maturation and activation marker CD80 along with the earlier marker of the immune response, CD86 (Figure 1C). Furthermore, the systemic induction of cytokines in the serum was analysed using ELISA (Figure 1D). The results indicated that after a 2-h treatment, SZU-101 induced the production of TNF-α to approximately 400 pg/ml, IFN-γ to approximately 150 pg/ml, IL-6 to 6,000 pg/ml and IL-12 to approximately 600 pg/ml, all of which were almost undetectable in the serum of PBS-treated control mice. After a 4-h treatment, the concentration of TNF-α decreased to basal levels, and the concentration of IL-6 also decreased by nearly 20-fold, while the concentrations of T_H_1 cytokines IFN-γ and IL-12 increased by approximately 1-fold each.

**Figure 2:**
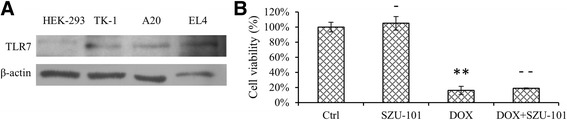
**SZU-101 exhibited no direct effect on EL4 cells. (A)** Endogenous TLR7 expression was confirmed in the murine lymphoma cell lines TK-1, A20 and EL4. **(B)** An MTT assay was performed to investigate the direct effect of SZU-101 on EL4 cell growth. (−*p* > 0.05 compared with control; **p < 0.01 compared with control; − −*p* > 0.05 compared with DOX; one-way ANOVA).

To confirm the expression of TLR7 in EL4 mouse lymphoma cells, an anti-TLR7 antibody was used to perform the Western blot detection, and murine lymphoma cells TK-1 and A20 were introduced as controls. The results indicated that EL4, TK-1 and A20 expressed endogenous TLR7, which was not detectable in HEK-293 negative controls (Figure 2A). An MTT assay was also performed to examine whether SZU-101 had a potential direct effect on tumour cells (Figure 2B). The result revealed that SZU-101 did not directly affect the growth of EL4 cells. The application of DOX significantly reduced EL4 cell viability, while the combined application with SZU-101 did not show a significant effect.

### SZU-101 in combination with DOX improves survival in a murine model of T cell lymphoma

**Figure 3:**
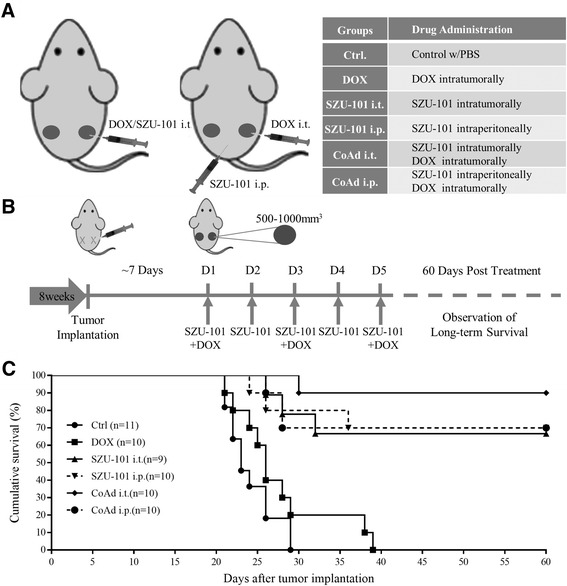
**Treatment schedule and survival curve. (A)** The left cartoon shows the administration methods of the drugs in each treatment group (listed in the right table). **(B)** The schematic diagram indicated the course schedule, in which the SZU-101 was administered daily from day 1 to day 5, and DOX was administered on days 1, 3 and 5. **(C)** The survival curve indicates 60-day survival post treatment of the tumour-bearing mice with different treatments. Each treatment group in the experiment contained at least nine mice.

Tumour-bearing mice were grouped into six treatment groups. As shown in Figure 3A, DOX was intratumourally administered, and SZU-101 was administered either intratumourally or intraperitoneally. The combined administration was performed by injecting both SZU-101 and DOX intratumourally or by injecting DOX intratumourally and injecting SZU-101 intraperitoneally. As indicated in Figure 3B, 8-week-old mice were implanted with EL4 cells, and the treatments began on the day when the tumour grew to 500–1,000 mm^3^ (day 1, D1). After one course of treatment, the mice were observed for drug effectiveness and long-term survival.

The first deaths were observed in the control group and DOX group on day 21 (Figure 3C). After a 60-day observation, mice treated with SZU-101 showed a substantial increase in long-term survival (defined as mice surviving greater than 60 days) compared with that of the control or DOX groups. Local administration of SZU-101 in combination with DOX moderately improved survival over other SZU-101 treatment groups (*p* < 0.01, log-rank test).

### Locally administered SZU-101 in combination with DOX led to a systemic antitumour effect

Tumour-bearing mice were implanted with two tumours on their backs, one on the left side and one on the right side. As depicted in Figure 3A, all intratumourally administered drugs were applied to the right-side tumours, whereas the left-side tumours were kept untreated to evaluate the systemic effect of treatment. Tumour volumes were measured as described above until tumours in any group were undetectable by the naked eye.

**Figure 4:**
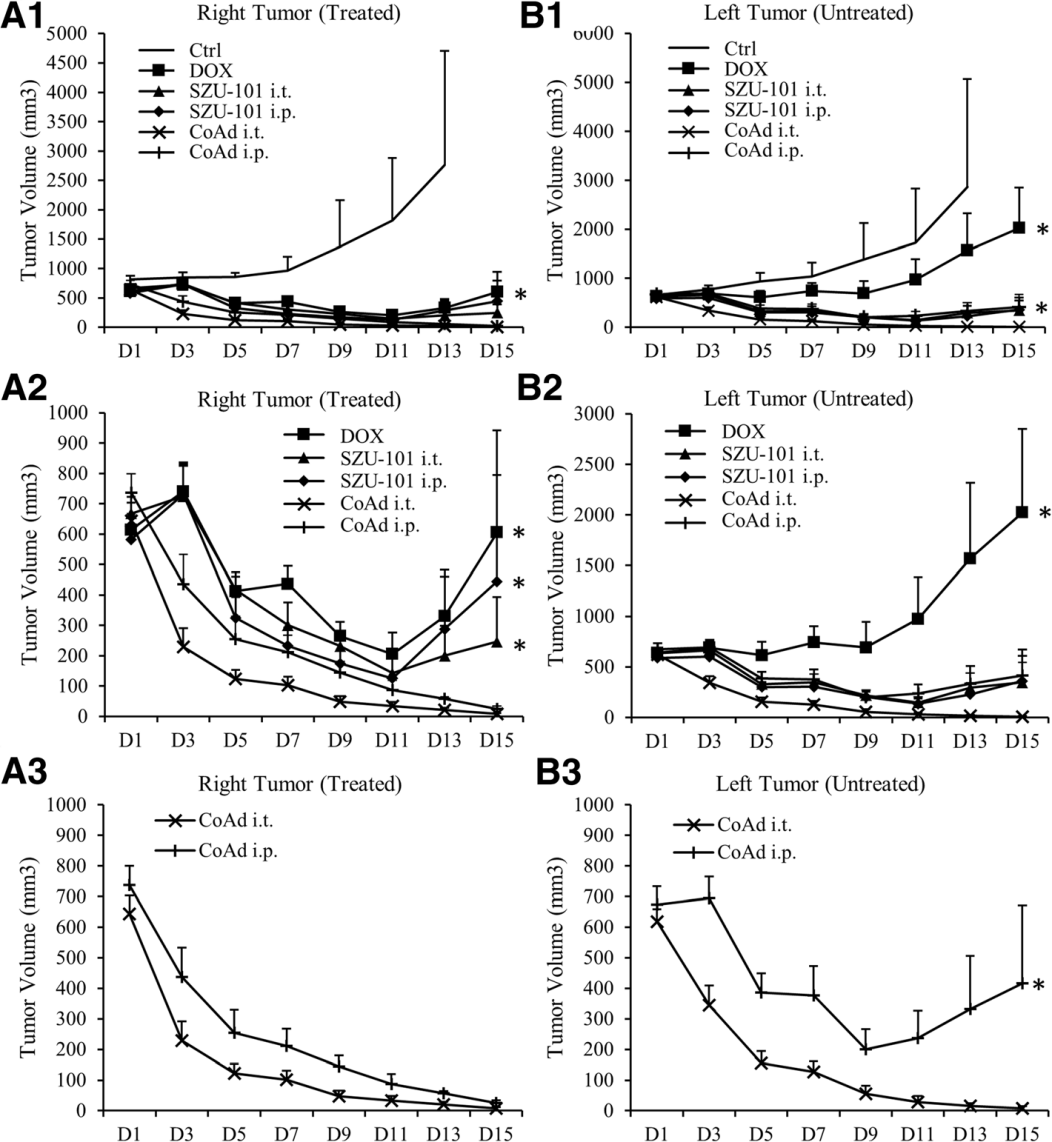
**Therapeutic effectiveness of combined treatment. (A1–3)**. The antitumour effect of different treatments on the directly treated right tumours. In **(A2)**, the PBS control was excluded, and in **(A3)**, only the combined treatments were compared. (**(A1)**, *p < 0.05 compared with PBS control; **(A2)**, *p < 0.05 compared with combined treatments; one-way ANOVA) **(B1–3)** The antitumour effect of different treatments on the untreated left tumours. In **(B2)**, the PBS control was excluded, and in **(B3)**, only the combined treatments were compared. (**(B1)**, *p < 0.05 compared with PBS control; **(B2)**, *p < 0.05 compared with SZU-101 treatments; **(B3)**, *p < 0.05 compared with CoAd i.t.; one-way ANOVA). Each treatment group contained at least nine mice.

All drug treatments showed a significant antitumour effect on the right-side tumours compared with PBS controls (Figure 4A1). The combined treatments (either combined administration intratumourally (CoAd i.t.) or CoAd intraperitoneally (i.p.)) exhibited improved tumouricidal effectiveness compared with single-drug treatments (SZU-101 i.t., SZU-101 i.p. or DOX), after which the tumours began to re-grow on day 11 (Figure 4A2). No significant difference was observed between the CoAd i.t. group and CoAd i.p. group (Figure 4A3).

On the left-side tumour, DOX treatment showed a moderate suppressive effect compared with PBS treatment (Figure 4B1), while the tumours in the DOX-treated group grew substantially larger than those in other treatment groups (SZU-101 i.t., SZU-101 i.p., CoAd i.t. or CoAd i.p.), especially after day 9 (Figure 4B2). Compared with the tumours in the CoAd i.p. group, the tumours in the CoAd i.t. group were significantly suppressed, and on day 15, the tumours were undetectable by the naked eye. Meanwhile, we also observed that the tumours in the CoAd i.p. group began to re-grow after day 9 (Figure 4B3).

### Long-term surviving mice generated an enhanced immune response to tumour cells

After 60 days of observation for long-term survival, surviving mice in the SZU-101 i.t., SZU-101 i.p., CoAd i.t. and CoAd i.p. treatment groups and normal untreated mice were sacrificed, and lymphocytes from each mouse were collected as effector cells. The effector cells were subsequently co-cultured with EL4 mouse T cell lymphoma cells at an effector:target cells ratio of 10:1.

After a 24-h co-culture, the medium was collected and centrifuged to remove the insoluble components. The supernatant was subsequently assayed for cytokine production by ELISA. The results indicated that the lymphocytes collected from CoAd i.t.-treated surviving mice generated a higher level of IFN-γ, which was approximately tenfold compared with control and two- to threefold compared with that from the SZU-101 i.t., SZU-101 i.p. or CoAd i.t. groups. Furthermore, IL-6 production was only observed from groups of intratumourally administered SZU-101, where a threefold increase in IL-6 production was detected from the CoAd i.t. group compared with that from the SZU-101 i.t. group.

**Figure 5:**
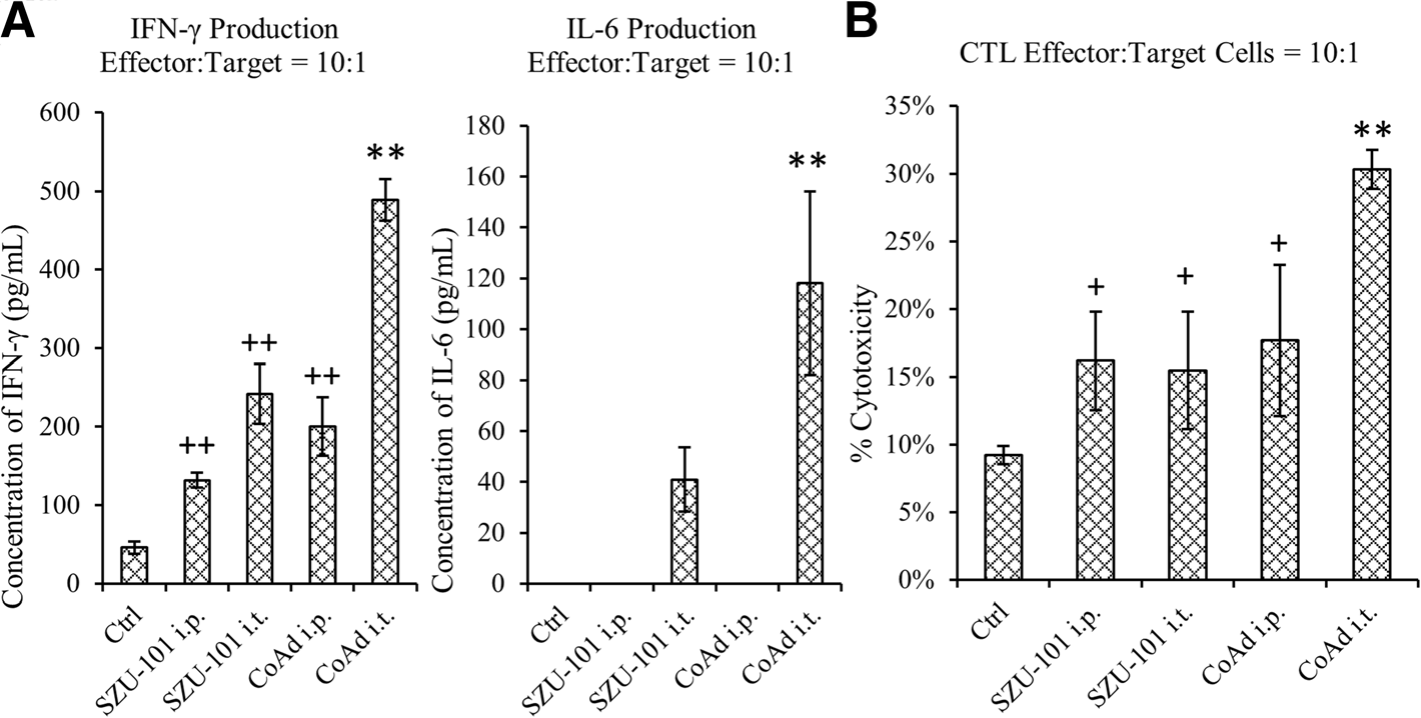
**The immune response by co-culture of murine lymphocytes with tumour cells.** The lymphocytes from long-term surviving mice were co-cultured with EL4 cells at an effector: target ratio of 10:1. **(A)** The cytokine induction of IFN-γ or IL-6 (IFN-γ production, ++*p* < 0.05 compared with control; **p < 0.05 compared with other SZU-101 treatments; IL-6, **p < 0.05 compared with SZU-101 i.t.; one-way ANOVA). **(B)** CTL-mediated cytotoxicity was assayed using the lymphocytes from mice with different treatments (+*p* < 0.05 compared with control; **p < 0.05 compared with other SZU-101 treatments; one-way ANOVA). Each group in the experiment contained at least six mice.

Moreover, co-cultured cells were also utilised to assess the cytotoxic T lymphocyte (CTL)-mediated cytotoxicity. The results indicated that effector cells from the CoAd i.t. group were significantly sensitive to EL4 tumour cells, while the effector cells from the SZU-101 i.t., SZU-101 i.p. and CoAd i.p. groups exhibited no intragroup significance (Figure 5B). The cytotoxicity to EL4 tumour cells in the control group was as low, as expected.

## Discussion

Lymphoma is a type of malignant cancer in the lymphohematopoietic system, presenting as a solid tumour in lymph nodes, which is called nodal lymphoma, or as extranodal lymphoma in the lymphoid tissues of many organs, such as the spleen, bone marrow, skin, brain, liver and bowels. Recent studies have revealed that combined immunotherapy for lymphoma may help overcome the suppressive micro-environment, induce tumour-specific immune response and improve tumour clearance.

It was reported that the administration of a conventional chemotherapeutic agent, such as cyclophosphamide or DOX, in combination with TNF-α, enhanced the antitumour effects against large established tumours in a murine lymphoma model [11,12]. The standard cyclophosphamide, DOX, vincristine and prednisone (CHOP) therapy was also used in combination with a monoclonal antibody, rituximab (R-CHOP), for CD20-positive B cell lymphoma, or with bryostatin 1 for human diffuse large cell lymphoma [13,14]. On the other hand, for immunotherapy, conventional antitumour therapy, such as monoclonal antibodies, chemotherapeutic agents and radiotherapy, was applied in combination with TLR agonists, such as TLR9 agonist 1018 ISS, TLR9 agonist CpG DNA 1826 and TLR7 agonist R848 [8-10]. These findings suggested that combined therapy with conventional drugs at a carefully selected dose and course schedule could lead to advanced antitumour effectiveness in lymphoma treatment. In this study, we focused on establishing a therapeutic schedule with the proper dose for the treatment of lymphoma using a TLR7 agonist synthesised in our lab combined with chemotherapeutic drugs in a murine T cell lymphoma model.

As the TLR7 was localised intracellularly, we introduced a fluorescence imaging technique and found that the synthesised compound can be transported to the cytoplasm (Additional file 2: Figure S2). We also demonstrated that SZU-101 can activate TLR7-NF-kB signalling in a TLR7-specific system at a low concentration of 1 μM after 6 h of stimulation or at a higher concentration of 5 μM after 4 h of stimulation. These findings suggested that SZU-101 is a TLR7 agonist and can induce downstream signalling through TLR7 activation.

It was reported that TLR7 activation could lead to strong T_H_-1-biassed immune responses and induce the release of proinflammatory cytokines [7,15,16]. In our study, we observed a high level of proinflammatory cytokines, such as TNF-α, IFN-γ and IL-12 in sera from SZU-101-treated tumour-bearing mice, while no induction of T_H_-2 cytokines IL-4, IL-5 or IL-10 was detected (Figure 1D). We also observed that one T_H_-2-biassed cytokine, IL-6 was significantly induced after agonism, which was in line with others’ reports that the activation of TLR7 by R848, 852A and imiquimod led to the release of the cytokines observed above through the TLR7-MYD88 pathway [17,10,18]. The T_H_-1 cytokines IFN-γ and IL-12 were significantly induced after 4-h treatment. However, more importantly, all induced cytokine productions were reduced to the basal level after 24-h treatment, providing a critical clue for planning the treatment schedule. Furthermore, TLR7 agonists could also significantly up-regulate the activation marker CD69 on B/T cells and activate dendritic cells [16,10]. In the study, we also found that the application of SZU-101 significantly induced the expression of the activation marker CD69 on B/T cells and stimulated the activation of dendritic cells (Figure 1C). These findings indicated that SZU-101 is a functional TLR7 agonist that can induce a strong immune response in tumour-bearing mice with such high effectiveness that the effect of SZU-101 on cytokine release and activation of immune cells was observed as early as 2 h after treatment.

In human lymphoma, the expression of TLR7 has been demonstrated in several studies. TLR7 was widely expressed in human follicular lymphoma (FL), diffuse large B cell lymphoma (DBCL) and peripheral T cell lymphoma (PTCL) as well as in other lymphocytic diseases, such as chronic lymphocytic leukaemia (CLL) [19-21]. In this study, as most of the murine lymphoma cells expressed endogenous TLR7 (Figure 2A), we sought to investigate whether SZU-101 had a direct effect on the tumour cells. The MTT assay results indicated that SZU-101 had no direct effect on EL4 tumour cells (Figure 2B), which was in line with others’ findings of another TLR7 agonist, imiquimod, on acute myeloid leukaemia (AML) cells, whereas the TLR7/8 agonist R848 may lead to terminal differentiation and suppressed cell proliferation in AML [22].

For lymphoma treatment, the low immunogenicity and immunosuppression have been the most problematic issues in the development of anticancer therapies. As described above, the combined application of conventional antitumour therapies with immune stimuli may provide potential strategies for lymphoma treatment. For instance, for B cell lymphoma, the TLR1/2 agonist (Pam_3_CSK_4_) was applied with the conventional chemotherapeutic agent Ara-C, resulting in a synergistic anticancer effect through the up-regulation of immunomodulatory molecules [23].

In our murine model of T cell lymphoma, the application of a conventional chemotherapeutic agent, DOX, which is a component of CHOP therapy, in combination with TLR7 agonist SZU-101, significantly improved survival compared with PBS treatment or DOX treatment alone. However, based on the survival data, no significance difference was observed between SZU-101-treated groups within 60 days post treatment (log-rank test, Figure 3C).

As the anticancer immunotherapy of TLR agonists could lead to the quick release and systemic dispersion of proinflammatory cytokines, the dose and administration method must be carefully selected [24]. To determine the treatment schedule and administration method, we chose a relatively low dose of DOX (3 mg/kg) and applied DOX every 2 days, such that the total usage of DOX was approximately 50% less than a previous study on CHOP therapy in a murine model of lymphoma [14]. Regarding the administration method, it was reported that the local administration of the TLR7 agonist imiquimod with anti-CD40 immunotherapy exhibited a strong antitumour effect in a murine model of malignant mesothelioma [25]. However, others have reported that the systemic administration of R848 with radiotherapy primed durable antitumour immune responses in a murine model of lymphoma [10]. In this study, we sought to include both local and systemic administration of SZU-101 to investigate the proper administration method for the combined therapy.

We generated a murine tumour model, in which two tumours were implanted on the same mouse to assess the local and systemic antitumour effect of the treatments. On the local tumour, which was directly treated with the drugs, the combined therapy led to significant improvement of the tumouricidal effect, especially 1 week post treatment, whereas the tumours in other groups began to re-grow (Figure 4A). However, in the distant tumour, which was not directly treated, DOX showed relatively low effectiveness compared with other treatments. We also observed that only DOX in combination with intratumourally administered SZU-101 (as local administration) exhibited a durable antitumour effect on the distant tumours, while tumour re-growth was noted in other groups of treatments, including the treatment of DOX in combination with intraperitoneally administered SZU-101 (as systemic administration; Figure 4B). These findings suggested that combined therapy with DOX and SZU-101 by local administration exhibited the best therapeutic performance in the murine model of EL4 T cell lymphoma by generating a strong local antitumour effect and inducing a systemic immune response against a distant tumour.

As the major effect of the activation of TLR7 is the induction of IFN-γ [18], we observed that when EL4 tumour cells were co-cultured with lymphocytes from the surviving mice treated by SZU-101, especially with the lymphocytes from the CoAd i.t. group, IFN-γ release was significantly increased (Figure 5A). We also found increased IL-6 induction when EL4 cells were co-cultured with lymphocytes from intratumourally administered SZU-101-treated mice (Figure 5A), suggesting that the intratumourally administered SZU-101 may have triggered an IL-6-based tumour-specific memory immune response. Furthermore, it was reported that the activation of TLR1/TLR2 signalling by a synthetic bacterial lipoprotein exhibits potential antitumour capabilities of up-regulating CTL function [26]. In this study, we found a similar result for TLR7, which suggested that the CTL function of the lymphocytes from SZU-101-treated surviving mice was significantly increased. In fact, lymphocytes from the CoAd i.t. group exhibited a 200% enhancement in CTL function (Figure 5B). Although the underlying mechanisms of the enhanced antitumour immune response by this combined therapy are unclear, based on the current data, we speculate that intratumourally administered SZU-101 enhances the process of antigen presentation on the tumour site where the tumour-specific antigens were concentrated by DOX-induced tumour cell death, while simultaneously activating immune cells to generate a durable systemic antitumour effect.

## Conclusions

In this study, we demonstrated for the first time that intratumourally administered TLR7 agonist SZU-101 can enhance the antitumour effectiveness of a conventionally used chemotherapeutic agent, DOX. The novel combination of intratumourally administered SZU-101 and DOX generated strong production of proinflammatory cytokines and enhanced the CTL response, leading to the eradication of both local and distant tumours in the murine model of T cell lymphoma. The important contribution made in this study was the demonstration of the successfully selected dose and course schedule, which may lower the side effects of chemotherapeutic drugs. We cannot omit the fact that the intratumoural administration of drugs to non-cutaneous tumours is often challenging, but our research still suggested that this combined immunotherapy can be developed as a promising treatment for lymphoma, which may further improve the effectiveness of the current standard CHOP therapy.

## Methods

### Mice, cell lines and animal model

The C57BL/6 mice used in this study were purchased from Guangdong Medical Laboratory Animal Center (Guangdong, China). The EL4 T cell lymphoma cells were maintained in DMEM (Gibco) with 10% FBS and 100 U/ml penicillin-streptomycin.

The C57BL/6 mouse lymphoma model was developed through the subcutaneous implantation of EL4 cells. Briefly, 1 × 10^6^ EL4 cells were inoculated subcutaneously on day 0 of each experiment. The mice were ready for experiments on day 7, when the tumours were approximately 500–1,000 mm^3^; the tumour volume was calculated using the following equation: [tumour volume = short axis^2^ × long axis/2] [27]. A visible tumour size of 20 mm in diameter was defined as the endpoint criterion, and those mice that met the criterion were sacrificed according to the AVMA guidelines on euthanasia.

All animal experiments were performed with the approval of Laboratory Animal Welfare and Ethics Committee, School of Medicine, Shenzhen University.

### Western blotting

The anti-TLR7 antibody was purchased from Santa Cruz Biotechnology and was applied to confirm TLR7 expression in the mouse lymphoma cell lines EL4, TK-1 and A20. Cell lysates were subjected to electrophoresis using 10% SDS-PAGE gels and transferred to PVDF membranes. The membranes were incubated with primary and secondary antibodies with proper blocking procedures and finally exposed to X-ray film (Fuji).

### HEK-BLUE assay

The TLR7 agonist SZU-101 was synthesised as described in Additional file 2: Figure S2. HEK-BLUE hTLR7 cells were purchased from InvivoGen. The cells stably expressed human TLR7 and a SEAP reporter, which can be used to detect TLR7 agonism through the activation of NF-kB signalling. The cells were maintained in selective DMEM growth medium with an additional 10 μg/ml blasticidin and 100 μg/ml Zeocin™. After incubation with different doses of SZU-101, the cells were tested using the HEK-BLUE detection kit according to the manufacturer’s instructions. The TLR7 agonist imiquimod and purified mouse TNF-α were used as positive controls. The induction of TLR7 activation can be visualised and assessed by reading the OD at 620–655 nm.

### MTS assay

A CellTiter® 96 MTS assay (Promega) was performed according to the manufacturer’s instructions. Briefly, 1 × 10^4^ EL4 cells were seeded in each well with PBS, SZU-101 (3 μg/μl), DOX (2 μg/μl) or SZU-101 combined with DOX. Cell viability was evaluated after a 24-h incubation.

### Flow cytometry

On day 7, the tumour-bearing mice were treated with 3 mg/kg SZU-101 or 4 mg/kg DOX by intraperitoneal administration. Mouse spleens were collected at 2, 4 or 24 h post treatment on day 8, and the splenocytes were prepared by removing the red blood cells with RBC lysis buffer (BioLegend) after separating the cells through a 70-μm cell strainer. Approximately 1 × 10^6^ cells were stained with corresponding florescence antibodies and analysed by FACSCalibur flow cytometry (BD Biosciences). The antibodies for flow cytometry were purchased from BioLegend and included the following: anti-CD11c-Alexa488, anti-CD86-PerCP/Cy5.5, anti-CD80-Alexa647, anti-CD3-Alexa488, anti-CD19-Alexa647 and anti-CD69-PerCP/Cy5.5.

### ELISA

The tumour-bearing mice were intraperitoneally administered SZU-101 (3 mg/kg) or DOX (4 mg/kg) on day 7. Sera samples were collected at 2, 4 or 24 h post treatment on day 8 and stored at −20°C. An ELISA for multiple cytokines in peripheral blood was performed using a Ready-SET-Go!® ELISA kit (eBioscience) according to the manufacturer’s instructions.

### Drug administration and treatment schedule

EL4 lymphoma cells were inoculated subcutaneously at two sites on the back of each mouse (approximately 20 g in weight) on the left and right side. The SZU-101 stock was prepared in DMSO at a concentration of 60 μg/μl, and DOX was prepared in PBS at a concentration of 2 μg/μl. The drugs for the mice in each group were prepared as described in Table 1.

**Table 1:** **Dose of SZU-101 and DOX in drug treatments**

**Group**	**Final con. and dose of SZU-101**	**Final con. and dose of DOX**	**Injection i.t. (drug/PBS)**	**Injection i.p. (drug/PBS)**
Ctrl	-	-	-/50 μl	-/100 μl
DOX	-	1.6 μg/μl; 4 mg/kg	50 μl/-	-/100 μl
SZU-101 i.t.	1.2 μg/μl; 3 mg/kg	-	50 μl/-	-/100 μl
SZU-101 i.p.	0.6 μg/μl; 3 mg/kg	-	-/50 μl	100 μl/-
CoAd i.t.	1.2 μg/μl; 3 mg/kg	1.6 μg/μl; 4 mg/kg	50 μl/-	-/100 μl
CoAd i.p.	0.6 μg/μl; 3 mg/kg	1.6 μg/μl; 4 mg/kg	50 μl/-	100 μl/-

The combined administration was performed by injecting SZU-101 and DOX intratumourally (CoAd i.t.) or injecting DOX intratumourally, while injecting SZU-101 intraperitoneally (CoAd i.p.).

DOX was applied on days 1, 4 and 7 of each course of treatment, and SZU-101 was applied every 24 h. A total of seven applications of SZU-101 and three applications of DOX were applied for one course. Tumour growth was measured daily, and long-term survival was evaluated.

### Cytotoxicity assay

Tumour-bearing mice treated with different drugs or combinations were sacrificed, and lymphocytes from each mouse were collected. The effector cells were incubated with EL4 mouse T cell lymphoma cells at an effector:target cell ratio of 10:1. The cytotoxicity experiment was performed using the CytoTox 96® Non-Radioactive Cytotoxicity Assay Kit according to the manufacturer’s instructions.

### Statistical analysis

Each group of assay included at least five samples for *in vitro* assays or ten mice for *in vivo* assays (except one mouse died in SZU-101 i.t. group). Statistical analysis was performed using SPSS 16 (IBM) and data was shown as mean ± standard error of the mean (SEM). Data was analysed by one-way ANOVA and a value of *p* < 0.05 was considered statistically significant.

## Additional files

Additional file 1: Figure S1. Fluorescein isothiocyanate (FITC) was purchased from AMRESCO and was conjugated to SZU-101. The HEK-293 cells were treated with unconjugated FITC or FITC-labelled SZU-101. The cells were incubated for 30 min at 37°C protected from light. The cells were washed twice with PBS and subsequently visualised by fluorescence microscopy. Additional file 2: Figure S2.
*A*. The schematic diagram indicated the synthesis method for SZU-101. Compound 1 was synthesised according to Wu’s methods [24] and was subsequently hydrogenated with R-Ni as catalyst, under 1 atm hydrogen and room temperature for 12 h. The filtered reaction mixture was concentrated by vacuum concentrator and purified by silica chromatography. The structure of Compound 2 was confirmed by ^1^H NMR and ^13^C NMR (*B*, *C*). Compound 2 was dissolved in CH_2_Cl_2_ and mixed with one equivalent mole of succinic anhydride. The mixture was stirred at RT for 12 h and heated at 60°C for 1 h. The reaction mixture was cooled to room temperature and concentrated. The concentrated residue was dissolved in 10% HCl and stirred overnight at room temperature to obtain compound SZU-101. The structure of SZU-101 was confirmed by ^1^H NMR and ^13^C NMR (*D*, *E*).

## Abbreviations

TLRs: Toll-like receptors
DOX: Doxorubicin
CHOP: Cyclophosphamide
DOX: vincristine and prednisone
CoAd: Combined administration
i.t: Intratumourally
i.p: Intraperitoneally
