# Supplementary figures and images for: Local administration of a novel Toll-like receptor 7 agonist in combination with doxorubicin induces durable tumouricidal effects in a murine model of T cell lymphoma
Source: J Hematol Oncol. 2015 Mar 4;8:21. doi: 10.1186/s13045-015-0121-9 (PMC4359787; doi:10.1186/s13045-015-0121-9)

Additional file 1

Figure S1


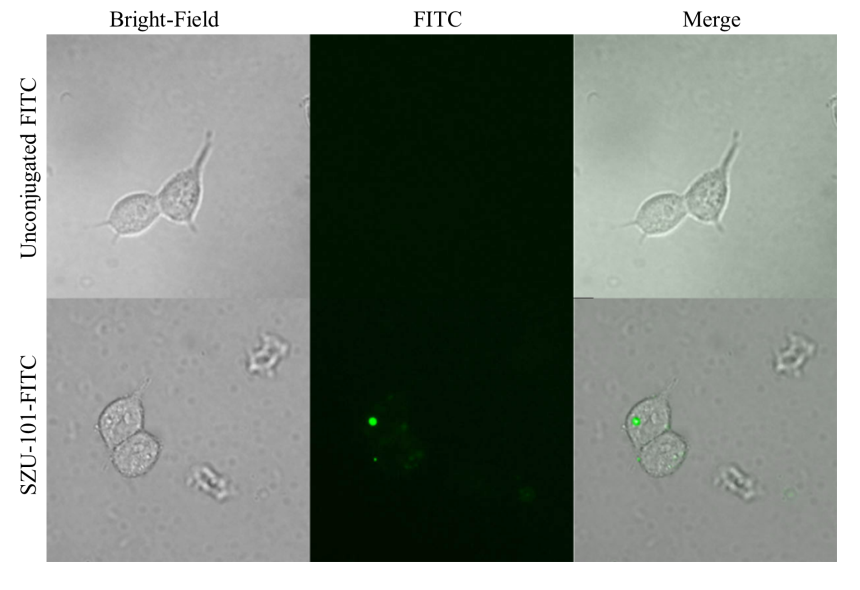

Supplement: Additional file 1: Figure S1. — Fluorescein isothiocyanate (FITC) was purchased from AMRESCO and was conjugated to SZU-101. The HEK-293 cells were treated with unconjugated FITC or FITC-labelled SZU-101. The cells were incubated for 30 min at 37°C protected from light. The cells were washed twice with PBS and subsequently visualised by fluorescence microscopy. [file 13045_2015_121_MOESM1_ESM.doc]

Additional file 2

Figure S2


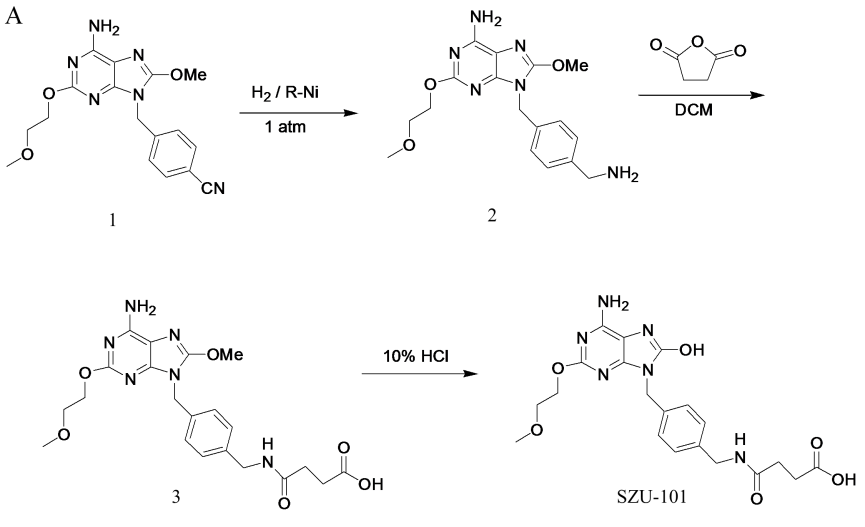


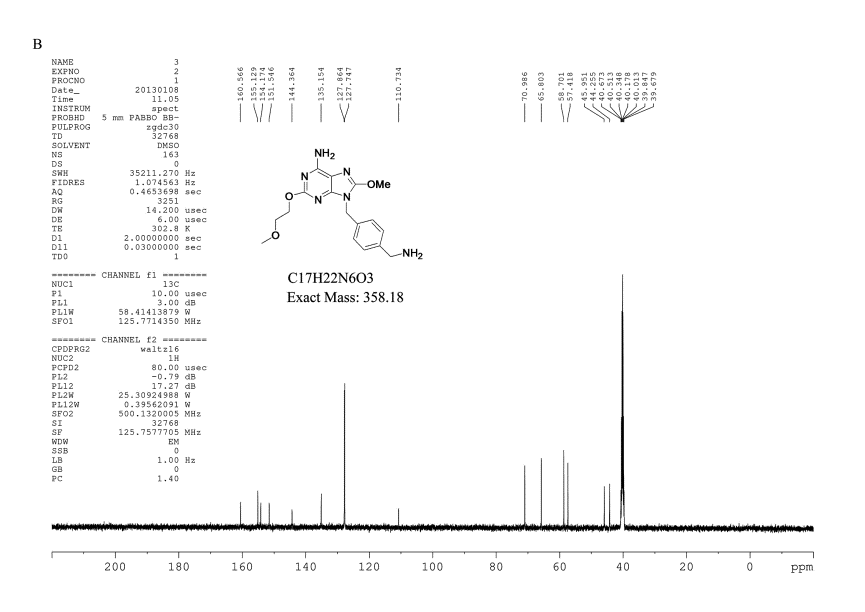


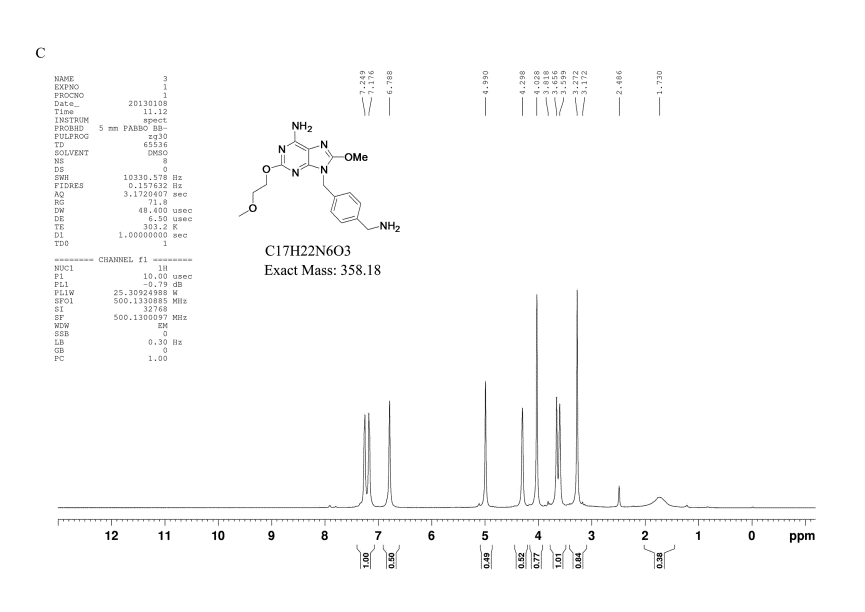


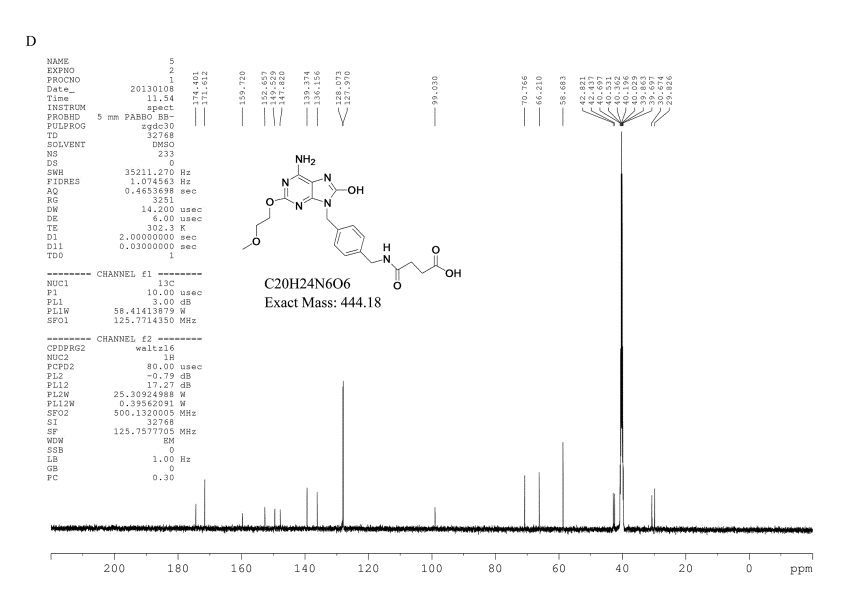


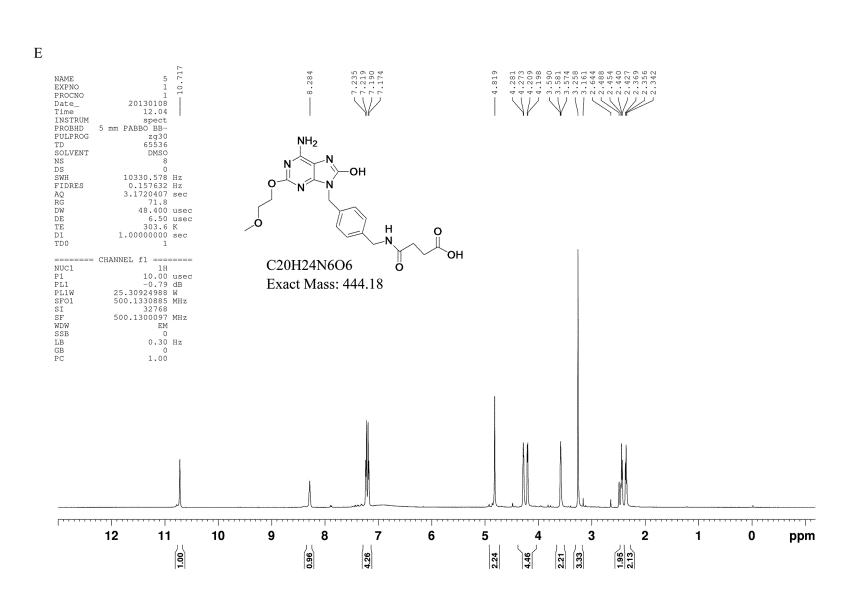

Supplement: Additional file 2: Figure S2. — A. The schematic diagram indicated the synthesis method for SZU-101. Compound 1 was synthesised according to Wu’s methods [24] and was subsequently hydrogenated with R-Ni as catalyst, under 1 atm hydrogen and room temperature for 12 h. The filtered reaction mixture was concentrated by vacuum concentrator and purified by silica chromatography. The structure of Compound 2 was confirmed by 1H NMR and 13C NMR (B, C). Compound 2 was dissolved in CH2Cl2 and mixed with one equivalent mole of succinic anhydride. The mixture was stirred at RT for 12 h and heated at 60°C for 1 h. The reaction mixture was cooled to room temperature and concentrated. The concentrated residue was dissolved in 10% HCl and stirred overnight at room temperature to obtain compound SZU-101. The structure of SZU-101 was confirmed by 1H NMR and 13C NMR (D, E). [file 13045_2015_121_MOESM2_ESM.doc]
